# A chaos theory inspired, asynchronous two-way encryption mechanism for cloud computing

**DOI:** 10.7717/peerj-cs.628

**Published:** 2021-08-09

**Authors:** Ravinder Rao Peechara, Sucharita V

**Affiliations:** 1 Department of Computer Science, Koneru Lakshmaiah Education Foundation, Guntur, Andra Pradesh, India; 2 Department of Computer Science and Engineering, Koneru Lakshmaiah Education FoundationGuntur, Andra Pradesh, India

**Keywords:** Randomness validation, Data encryption, Higher order randomness, Chaos theory, Key generation, Event based

## Abstract

Data exchange over the Internet and other access channels is on the rise, leads to the insecurity of consequences. Many experiments have been conducted to investigate time-efficient and high-randomized encryption methods for the data. The latest studies, however, have still been debated because of different factors. The study outcomes do not yield completely random keys for encryption methods that are longer than this. Prominent repetition makes the processes predictable and susceptible to assaults. Furthermore, recently generated keys need recent algorithms to run at a high volume of transactional data successfully. In this article, the proposed solutions to these two critical issues are presented. In the beginning, one must use the chaotic series of events for generating keys is sufficient to obtain a high degree of randomness. Moreover, this work also proposes a novel and non-traditional validation test to determine the true randomness of the keys produced from a correlation algorithm. An approximate 100% probability of the vital phase over almost infinitely long-time intervals minimizes the algorithms’ complexity for the higher volume of data security. It is suggested that these algorithms are mainly intended for cloud-based transactions. Data volume is potentially higher and extremely changeable 3% to 4% of the improvement in data transmission time with suggested algorithms. This research has the potential to improve communication systems over ten years by unblocking decades-long bottlenecks.

## Introduction

The digital era has motivated education, research, and enterprises to communicate more on digital platforms. The communications restrict textual communication or information status updates. Instead, it has also included communications inclusive of the business data, which is critical for the organizations. Thus, the demand for security to protect the privacy of these datasets during transmission is also becoming highly important and challenging. Detailed research by Stallings (2011) has identified that the networking protocols can be highly beneficial. At the same time, they are highly vulnerable in the absence of the appropriate security measures. This demand for appropriate security mechanisms has motivated several researchers to contribute towards data security and the research outcomes of the encryption and decryption-based methodologies. The notable work by Dworkin (2007) has justified the demand for these research attempts. Also, it has furnished the guidelines for cipher block generations. The first outcome in this direction was generated by Bender et al. (1996) in early 1996 during the initial phases of data encryption research trends.

Furthermore, the trend has continued to grow as the higher demand for data security has emerged. Rather than only securing the textual formats, the demand for data security in rich multimedia formats has also emerged. Hence, the research trends can also be observed in steganography or data hiding in multimedia data formats. The work suggested by Cox et al. (2007) has significantly demonstrated the performance improvements over the vulnerabilities of higher volume data. In recent times, with the industrial growth of higher-performance computing, such as cloud computing, they have removed the barrier of computing capacities. They have given the freedom to deploy higher security mechanisms for large volumes of data. Henceforth, this advancement has motivated several researchers to carry forward complex algorithms to ensure data security. The recent outcome of the research by Johnson & Jajodia (1998) has proved that higher computing capacities can be highly beneficial for data security and the encryption and decryption methods.

Henceforth, this work presents two major bottlenecks of the data security research:

• True randomness of the security key generation;
• Validation of the generated keys with a higher level of vulnerability analysis.

With the bottleneck of limitations of computing capacities being removed.

The significant addition of this paper is as follows:

1. This work explores and designs complex security methods to generate the keys using the chaos theory.

2. Validation of the generated keys against vulnerabilities by introducing control set mechanism.

The rest of the work is organized as follows, In ‘Chaos Theory Fundamentals’, the fundamentals of chaos theory are explicated, and the applicability for key-generation is understood. In ‘Fundamentals of Encryption and Decryption Methods’, the fundamentals of the key-generation methods are understood. In ‘Parallel Research Outcomes’, the parallel research outcomes are analyzed critically. In ‘Problem Formulation’, the identified problem is formulated mathematically. In ‘Proposed Solution—The Mathematical Model’, the proposed solution is presented. In ‘Proposed Algorithms’, the proposed algorithms are furnished, in ‘Results and Discussions’, the obtained results are analyzed with comparative analysis. Finally, in ‘Conclusion’, the conclusion of the research paper is written.

## Chaos Theory Fundamentals

In this section of the work, the fundamental of chaos theory is understood with the encryption key generation’s applicability. The fundamental of the chaos theory defines the unpredictability of the sequences of events. In principle, the chaos theory is initially predictable. However, for a large set of outcomes, the outcomes eventually become unpredictable. Thus, generating random values for a considerable time duration for a considerable number of samples can be very effective, using the chaos theory outcomes.

The fundamental formulation for chaos theory can be presented as,

(1) \begin{eqnarray*}K(t+1)=R.[1-K(t)]\end{eqnarray*}

The *K*(*t* + 1) defines the current outcome, *K*(*t*) defines the previous outcome, and *R* denotes the situation’s randomness. The randomness of the chaos theory primarily relies on three factors:

• The amount of uncertainty or randomness is to be accomplished in the outcome.
• The randomness of the current outcome.
• It depends on the duration of the chaos or the outcome generation’s duration, or the number of outcomes generated.

Henceforth, the prior understanding of the chaos principle ensures a higher randomness range during the security key generation for encryption and decryption. Ensuring the following factors:

1. Firstly, the randomness coefficient, R, must be highly random to ensure higher order randomness of the generated keys.
2. Secondly, the generated keys at each step must be validated for randomness.
3. Finally, the random critical generation process must sustain a prolonged duration.

Thus, relying on these three parameters, the actual randomized key generation can be achieved. Further, in the upcoming sections of this work, building on this principle, the key-generation process is realized and elaborated with mathematical models. Nevertheless, before presenting the proposed method and the recent research outcomes, this work also furnishes the fundamental process of the key-generation in the next section.

## Fundamentals of Encryption and Decryption Methods

To have the fundamental understanding of the chaos theory and the applicability for the security key generation, in this section of the work, the fundamental algorithms are understood. Primarily, there are three major algorithms, which are the starting point of these directions of the research. The algorithms are Data Encryption Standard (DES), Advanced Encryption Standards (AES), and Rivest, Shamir and Adleman Standard (RSA).

Firstly Data Encryption Standard DES key generation method is understood:

**Table:** 

Step -1. Accept 64-bit plain text
Step -2. Generate 56-bit key
Step -3. Shift the plain text block parallel to the Key bits
Step -4. Remove parity bits from the key
Step -5. Split key into 28 sections
Step -6. For each
a. Rotate the keys
b. Combine the sections
c. Compression permutation to reduce the key from 56 bits to 48 bits

Secondly Advanced Encryption Standards AES key generation method is understood:

**Table:** 

Step -1. Derive the set of round keys from the cipher key.
Step -2. Initialize the state array with the block data (plaintext).
Step -3. Add the initial round key to the starting state array.

Finally Rivest, Shamir & Adleman Standard RSA key generation method is understood:

**Table:** 

Step -1. Select a value of e from a random set of prime numbers
Step -2. repeat
Step -3. p ← genprime(k/2)
Step -4. until (p mod e) ≠ 1
Step -5. repeat
a. q ← genprime(k - k/2)
Step -6. until (q mod e) ≠ 1
a. N ← pq
b. L ← (p-1)(q-1)
c. d ← mod Inv(e, L)
Step -7. return (N, e, d)

Thus, it is natural to realize that the following conditions bottleneck the fundamental methods:

• The generation of the keys for encryption and decryption for an infinite timeline is not unique.
• Validation of the randomness of the key generation process is coexisting with the actual key generation algorithm. Thus, the validation is invalid.

These claims are also proven in the other sections of this work. Henceforth, in the next section of this work, the recent improvements are discussed.

## Parallel Research Outcomes

After the detailed analysis of the chaos theory and the fundamentals of the key generation methods, the parallel research outcomes are analyzed and discussed in this section.

The security protocols for data encryption have always been bounded to have the reversibility of the encryption process. The importance of the reversibility capabilities of any encryption algorithm is demonstrated by Ni et al. (2006). Further, building on the same principle, Puech, Chaumont & Strauss (2008) have demonstrated that similar analogies must be addressed for any type and scale of the data. The work by Zhang (2011) in recent times also confirms a similar principle. Nevertheless, fundamental bottleneck of such strategies is the reversing, or the decryption process is highly dependent on the encryption process. The security keys are generated from a primary source, which can be highly vulnerable to attacks.

On the other hand, they build similar reversible strategies, Hong, Chen & Wu (2012) have showcased that different but dependable methods can be deployed for the key generation. This method opened a newer angle for researchers. Nonetheless, the complete autonomy for the key generation must be adopted with a higher randomness level for this purpose. Hence, the use of machine learning methods for generating reversible security methods is highly recommended. The work showcased by Manikandan & Masilamani (2018) confirms this claim.

The strategies for data security are the same irrespective of the nature of the size of the data. However, the distributed nature of the data must comply with newer data security protocols. The work by Qian & Zhang (2016) has confirmed this claim.

The encryption methodologies for any data primarily depend on the key generation methods. A good number of research attempts can be seen in the direction of the key generation. The work of Xiong, Xu & Shi (2018) has showcased the recent improvements for security key generations, using the wavelet transform method. Relying on a similar principle by Weng et al. (2019), it has been proven that these kinds of approaches are always applicable for the data irrespective of the size or distribution. In a similar research line, another outcome by Liu, Lu & Yan (2019) has been showcased using the polynomial-based key generation method. Nonetheless, this approach is criticized for the higher time complexity. The work by Tang et al. (2012) in previous times has proven that using a higher-level data structure, such as a tree, can reduce the time complexity to a greater extent. This strategy can also be observed in the work of many other researchers as Thakur & Omprakash (2018) and Alfalou et al. (2011) and Iwendi & Allen (2012).

On the other hand, a few research attempts can ensure the encryption and decryption process with the data’s compression. The notable work by Garima & Abhishek (2014) and Muhammad & Nordin (2016) have justified this belief. The fundamentals of these methods are well presented in Shunmugan & Arockia Jansi Rani (2016) and Jeeva, Palanisamy & Kanagaram (2012).

### Limitations of the current work

The work adopted the outcomes from the Chaos Theory, which is criticized for the following reasons:

Sensitivity to initial conditions as each point in a chaotic system is arbitrarily closely approximated by other points that have significantly different future paths or trajectories. Thus, an arbitrarily small change or perturbation of the current trajectory may lead to significantly different future behavior.

A chaotic system may have sequences of values for the evolving variable that exactly repeat themselves, giving periodic behavior starting from any point in that sequence. However, such periodic sequences are repelling rather than attracting, meaning that if the evolving variable is outside the sequence, however close, it will not enter the sequence, and, in fact, will diverge from it. Thus, for almost all initial conditions, the variable evolves chaotically with non-periodic behaviour.

Henceforth, with the detailed analysis of the parallel research attempts, the identified problems are formulated using mathematical modelling in the next section.

## Problem Formulation

After the detailed analysis of the parallel research attempts, the problems identified in the recent research outcomes are formulated using mathematical models. This section primarily focuses on two significant problems for the study’s advancements as a random key generation and the randomness validation of the generated keys.

Firstly, the problem regarding the random key generation is formalized. The problem with the existing approach is the genuine randomness of the generated public and private keys.

Lemma 1 The generation of the keys for encryption and decryption for an infinite timeline is not unique.

Proof Assuming that the key set, *K*[], is collecting the individual key items, *k*_*i*_, which is again an outcome from the time-dependent function. Thus, the primary relation, for a total of *n* number of observations, can be formulated as,

(2) \begin{eqnarray*}K[]=\sum _{i=1}^{n}{k}_{i}\end{eqnarray*}

Further, as the generated keys are time-dependent thus, each key can be presented as,

(3) \begin{eqnarray*}{k}_{i}\leftarrow \Phi (t)\end{eqnarray*}

The two different keys are generated as *k*1_*i*_ and *k*2_*i*_ on the time instances as *t*_1_ and *t*_2_. Thus this relation can be formulated as,

(4) \begin{eqnarray*}k{1}_{i}\leftarrow \Phi ({t}_{1})\end{eqnarray*}

And,

(5) \begin{eqnarray*}k{2}_{i}\leftarrow \Phi ({t}_{2})\end{eqnarray*}

These time instances are not the same, hence,

(6) \begin{eqnarray*}{t}_{1}\not = {t}_{2}\end{eqnarray*}

Nevertheless, the random value generation function, which is time-dependant, is bound to have one upper limit *λ*, during the key generation, as mentioned below:

(7) \begin{eqnarray*}\int \nolimits \nolimits _{1}^{\lambda }\Phi \rightarrow \Phi (t)\end{eqnarray*}

Eventually, the upper limit *λ* is bound to be reached for a longer time duration. For every *t*_*n*_ time instance, the upper limit is reached for this random key generation function, which again can be presented as,

(8) \begin{eqnarray*}\Phi (t)=\Phi ({t}_{n})\end{eqnarray*}

Thus, as the *t*_2_ time instance reaches the *t*_*n*_ time instance, the uniqueness and the randomness of the generated keys must repeat. The complete key generation process starts with generating the non-unique keys. As

(9) \begin{eqnarray*}\Phi (t): \left\{ \begin{array}{@{}l@{}} \displaystyle {t}_{1}\rightarrow {t}_{2}\rightarrow {t}_{n}\\ \displaystyle k{1}_{i}\approx k{2}_{i} \end{array} \right. \end{eqnarray*}

Henceforth, the uniqueness of the key generation process is violated and makes the key sets vulnerable to attacks.

Thus, this problem must be solved.

The problem is regarding the randomness validation of the generated keys. The primary issue with the existing methods is that the deployed method for validating the random keys relies on the key generation’s same principle. With both the methods being the same for randomness, the vulnerability cannot be justified.

Lemma 2 Validation of the randomness of the key generation process is coexisting on the actual key generation algorithm. Thus, the validation is invalid.

Proof Assuming that the key set, *K*[], is collecting the individual key items, *k*_*i*_, which is again an outcome from the time-dependent function. Thus, the primary relation, for a total of n number of observations, can be formulated as,

(10) \begin{eqnarray*}K[]=\sum _{i=1}^{n}{k}_{i}\end{eqnarray*}

Further, as the generated keys are time-dependent thus, each key can be presented as,

(11) \begin{eqnarray*}{k}_{i}\leftarrow \Phi (t)\end{eqnarray*}

Assuming that the key generation’s primary function is Φ_1_(*t*) andfor randomness validation, the used function is Φ_2_(*t*).

 Here, as both the functions have the same limits and terminating conditions with the same characteristics, it is safe to state that the functions are the same.

(12) \begin{eqnarray*}{\Phi }_{1}(t)={\Phi }_{2}(t)\end{eqnarray*}

Again, assuming that each function generates a set of keys as *K*1[] and *K*2[]. Thus, due to the similar characteristics of the functions, the generated random key sets also must be similar. Thus,

(13) \begin{eqnarray*}K1[]=K2[]\end{eqnarray*}

Henceforth, following Eq. (10), the following can be stated: each set must contain a similar set of values.

(14) \begin{eqnarray*}\prod _{i=n}{k}_{i}=\prod _{j=n}{k}_{j}\end{eqnarray*}

Thus, the random key generated pattern will be similar. The key randomness validation method will justify the primary key generation method. This validation will generate a confirmation for validation each time irrespective of the actual randomness of the elements. Hence, this problem also needs to be addressed and solved. Furthermore, with a detailed understanding of the problems of the mathematical models, the solution is realized using mathematical formulations in the next section of this work.

## Proposed Solution—The Mathematical Model

After a detailed understanding of the similar research outcomes and mathematical formulation of the problems, the proposed solutions are presented using the mathematical models in this section. Firstly, the key generation approach using the chaos theory is formulated with the proposed terminating condition.

Lemma 3 Generation of the keys for encryption and decryption using a two-phase chaos method terminating condition is highly random.

Proof Assuming that the key set, *K*[], collects the individual essential items, *k*_*i*_, results from the time-dependent function. Thus, the primary relation, for a total of *n* number of observations, can be formulated as,

(15) \begin{eqnarray*}K[]=\sum _{i=1}^{n}{k}_{i}\end{eqnarray*}

Depending on the basic foundation of the chaos theory, the function responsible for generating the random keys, *φ*, can be further formulated as,

(16) \begin{eqnarray*}{k}_{i}(t)=\varphi (t).[1-{k}_{i}(t-1)]\end{eqnarray*}

The randomness for the key generation function is again a time-dependent function, and further can be formulated as,

(17) \begin{eqnarray*}\varphi (t)\leftarrow \prod _{time=t}r[]\end{eqnarray*}

Nonetheless, like any other randomness generation function, this function *φ*(*t*)must have one upper limit. Beyond that specified upper limit, the function shall produce the repetition of the random values, which will make the generated keys vulnerable to attacks. As the random value generation function, which is time-dependant is bound to have one upper limit, *λ*, during the key generation, as,

(18) \begin{eqnarray*}\int \nolimits \nolimits _{1}^{\lambda }\varphi \rightarrow \varphi (t)\end{eqnarray*}

Henceforth, stopping the function for random value generation before the upper limit is a must to ensure randomness. Assuming that the upper limit, *λ*, shall be reached in a time limit of t _*n*_, thus, the function must be stopped within the t _*n*_ time limit. It can be formulated as,

(19) \begin{eqnarray*}{k}_{i}(t)= \left[ \prod _{\lim _{t\rightarrow {t}_{n}}}r[] \right] .[1-{k}_{i}(t-1)]\end{eqnarray*}

Thus, the generated key, *k*_*i*_(*t*), is ensured to have no repetitions and can be further used for public and private key component generation. It is Phase-I of the proposed key generation method.

Further, in Phase-II, the public key and the private key must be generated as Key _*Pub*_ and Key _*Pri*_ respectively. It can be formulated, using two instances of the *k*_*i*_(*t*) and *k*_*i*_(*t* + 1) at any random instance ∂, as,

(20) \begin{eqnarray*}Ke{y}_{Pub}={!}\prod _{Random=\partial }{k}_{i}(t)\wedge {k}_{i}(t+1)\end{eqnarray*}

And,

(21) \begin{eqnarray*}Ke{y}_{\Pr\nolimits i}={!}[\prod _{Random=\partial }{k}_{i}(t)\wedge {k}_{i}(t+1)]\end{eqnarray*}

Henceforth, the generation of the keys is completed and ensured to be highly random. This process is Phase-II of the fundamental generation mechanisms.

Secondly, the randomness of the generated keys must be validated to ensure security.

Lemma 4 Validation of the uniqueness of generated keys by introducing a control set can result in perfect validation.

Proof Assuming that the key set, *K*[], is a collection of the individual key items, *k*_*i*_, which is again an outcome from the time-dependent function.

 Thus, the primary relation, for a total of n number of observations, can be formulated as,

(22) \begin{eqnarray*}K[]=\sum _{i=1}^{n}{k}_{i}\end{eqnarray*}

Simultaneously, assuming that the key set, *K*1[], is a collection of the individual key items, *k*1_*i*_, which is again an outcome from the time-dependent function.

Thus, the primary relation, for a total of n number of observations, can be formulated as,

(23) \begin{eqnarray*}K1[]=\sum _{i=1}^{n}k{1}_{i}\end{eqnarray*}

Further, to validate the randomness of the generated keys, the standard deviation must be calculated for the generated key set and the control key *τ*_1_*τ*_2_. It can be formulated as,

(24) \begin{eqnarray*}\tau = \frac{dK[]}{dn} \end{eqnarray*}

And,

(25) \begin{eqnarray*}\tau 1= \frac{dK1[]}{dn} \end{eqnarray*}

Thus, the similarity measure, X, can be formulated as,

(26) \begin{eqnarray*}\mathrm{P}= \frac{(K[].{\tau }_{2})^{2}-(K1[].{\tau }_{1})^{2}}{{\tau }_{1}.{\tau }_{2}} \end{eqnarray*}

Hence, based on general correlation theory, if the *P*-value is less than 0.05, it is safe. To state that the generated keys are highly random and do not control the set keys.

 Henceforth, this section of the work formulates the random key generation’s mathematical models and randomness validation. In the next section of this work, based on the mathematical foundations, the proposed algorithms are furnished and discussed.

## Proposed Algorithms

The identified problems are formalized and the proposed solution is furnished in this section after the detailed analysis of the parallel research recent improvements. The proposed algorithms are furnished in this section.

This work primarily proposes two major algorithms, firstly for the random key generation and secondly for validating the proposed key generation method using a diversified randomness verifier.

Firstly, the proposed key generation algorithm is furnished.

**Table:** 

**Algorithm**-I: Two-Phase Chaos Coefficient-Based Security Key Generation (**TPCCSKG**) Algorithm
Step - 1. Initialize the first collection of the keys as *K*[]
Step - 2. Initialize the second collection of the randomness coefficient as *R*[]
Step - 3. Phase 1: For *n* instances,
i. Generate the random collection as *R*[*n* + 1] = Random(n)
ii. If, *R*[*n* + 1] = = *R*[*n*]
iii. Then, stop the random value generation
Step - 4. Phase 2: For *t* instances,
i. Generate the random key collection as *K*[*t* + 1] = *R*[*t*].(1 − *K*[*t*])
ii. If, *K*[*t* + 1] = = *K*[*t*]
iii. Then, stop the random key generation
Step - 5. Generate the public key, Pub_Key = !(Random Instance of K[t+1]) XOR K[t]
Step - 6. Generate the private key, Pri_Key = !(Random Instance of K[t+1] XOR K[t])
Step - 7. Return the Pub_Key & Pri_Key pair

The beginning conditions imply that each point in the framework has different focuses with extraordinary future directions.

A disordered framework may have series of qualities for developing the variable that precisely rework themselves, giving intermittent conduct beginning from any point in that arrangement. Nonetheless, such intermittent arrangements are repulsing instead of drawing. In implying that if the developing variable is outside the grouping, to close, it will not enter the succession and will veer from it. Hence, for practically all underlying conditions, the variable advances riotously with non-intermittent conduct.

Secondly, the proposed key validation algorithm is furnished.

**Table:** 

**Algorithm - II**: Trend Based Security Key Validation (**TBSKV**) Algorithm
Step - 1. Accept the series of generated Pub_Key as PK[]
Step - 2. Accept the series of generated Pri_Key as PVK[]
Step - 3. Generate the random trend sequence as T[]
Step - 4. Calculate the mean for PK[] as MPK
Step - 5. Calculate the mean for PVK[] as MPVK
Step - 6. Calculate the mean for T[] as MT
Step - 7. Calculate the correlation factor for PK[] as CPK
a. CPK ={(PK[].MT) ^2^ - (T[].MPK) ^2^}/(MPK.MT)
Step - 8. Calculate the correlation factor for PVK[] as CPVK
a. CPVK = (PVK[].MT)2 - (T[].MPVK)2/(MPVK.MT)
Step - 9. If CPK and CPVK as CPK! =CPVK and CPK, CPVK > 0.05 [As per correlation theory]
Step - 10. Then, Return the Pub_Key & Pri_Key pair
Step - 11. Else, drop the Pub_Key & Pri_Key pair

Connections are helpful because they can demonstrate a prescient relationship that can be abused practically speaking. For instance, an electrical utility may deliver less force on a gentle day, dependent on the connection between power interest and climate. There is a causal relationship in this model. An outrageous environment makes individuals utilize greater power for warming or cooling. In this model, the presence of a connection is not adequate to derive the presence of a causal relationship.

The data given by a connection coefficient is not enough to characterize the reliance structure between arbitrary factors. The connection coefficient characterizes the reliance structure in particular cases, for instance, when the circulation is an ordinary multivariate conveyance.

## Algorithm—I & II: Computational Complexity Analysis

For building the first *K*[] set of elements with *n* number of elements and *t* amount of unique time, the total time complexity, *t*1, can be formulated as,

(27) \begin{eqnarray*}t1=n.t=O(n.t)\end{eqnarray*}

The value, *t* is much less than the number of elements as *t* <  < *n* . Thus, the Eq. (1) can be re-written as,

(28) \begin{eqnarray*}t1=O(n)\end{eqnarray*}

Further, the second level of randomness coefficient can be generated in *t*2 time with m number of elements as,

(29) \begin{eqnarray*}t2=t.m=O(t.m)\end{eqnarray*}

Following the same principle as mentioned in Eq. (2), Eq. (3) can be re-written as,

(30) \begin{eqnarray*}t2=O(m)\end{eqnarray*}

Further, the total time, *T*, can be formulated as,

(31) \begin{eqnarray*}T=t1+t2=O(n)+O(m)\end{eqnarray*}

As, *n* = *m* , thus, Eq. (5) can be re-written as,

(32) \begin{eqnarray*}T=O(2n)\end{eqnarray*}

Henceforth, in the next section of this work, the results from these proposed algorithms are discussed.

## Results and Discussions

After the detailed analysis of the existing research outcomes, formulation of the problem, and identification of the proposed solutions, the obtained results are furnished and discussed in this section of the work.

The obtained results are highly satisfactory, and, in this section, the results are furnished in five segments.

### Experimental setup

**Figure 1:**
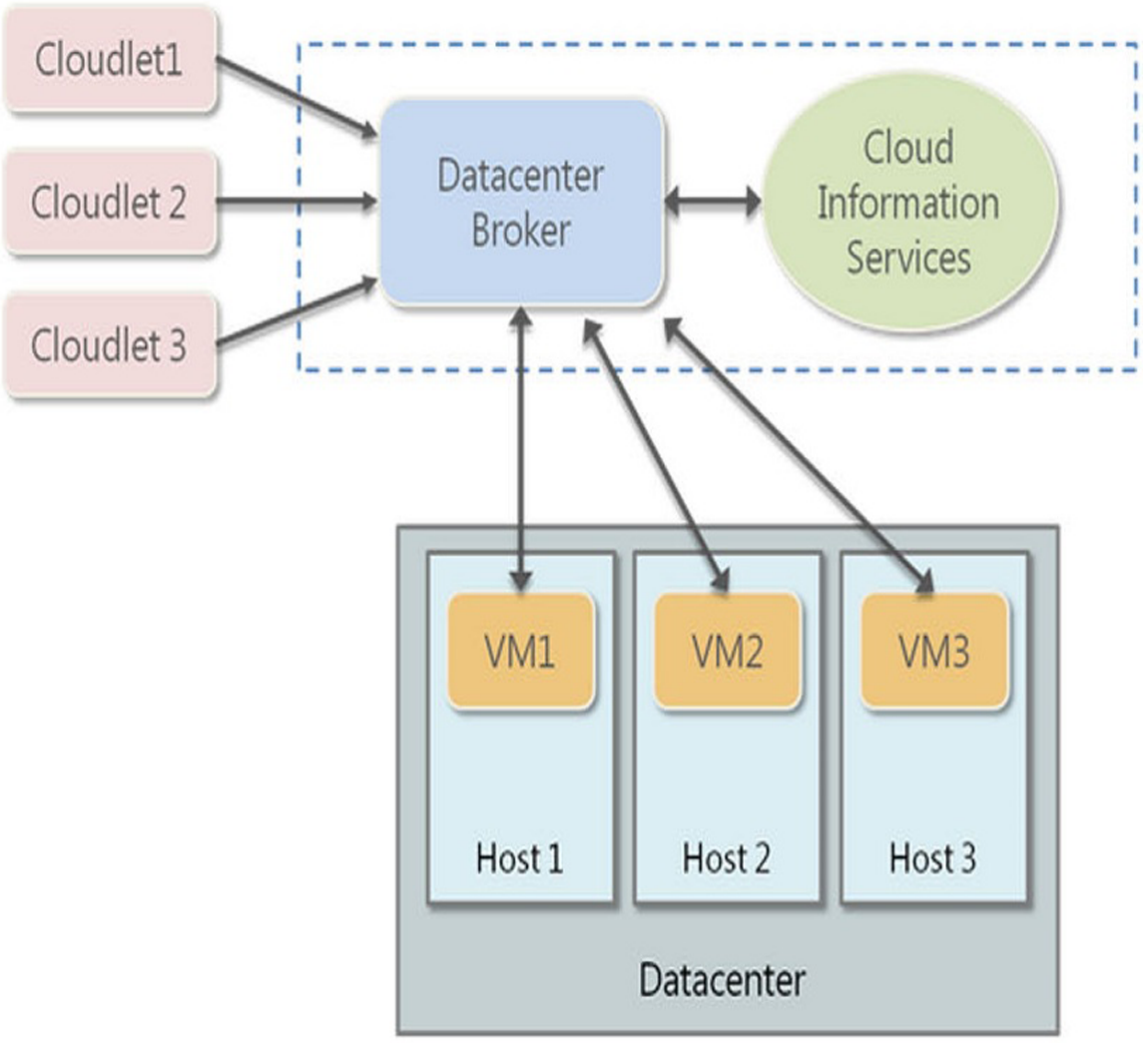
Cloud Sim Deployment Model.

Firstly, the experimental setup is analyzed here. The proposed algorithms are deployed on the CloudSim simulator for testing the encryption and decryption performances (Fig. 1).

The initial configuration is furnished here (Table 1).

**Table 1:** Experimental setup.

**Configuration parameter**	**Value**
Number of hosts	50
Number of VMs	50
Total simulation time	86400.00 s
Energy consumption	34.35 kWh
Number of VM migrations	2203
Number of Physical Host Start	685
Number of Physical Host Shutdown	685

CloudSim is a structure for displaying and recreation of distributed computing frameworks and strategies. Initially, in the Cloud Computing and Distributed Systems Laboratory, the University of Melbourne, Australia, CloudSim has gotten one of the most mainstream open-source cloud test systems in examination and the scholarly community. CloudSim is written in Java.

### Key generation results

Secondly, this work defines a novel approach for the generation of the keys. Hence, in this sub-section, the critical generation results are formulated (Table 2). The result is presented with 20 simulation outcomes.

**Table 2:** Key generation results.

**Simulation no#**	**Generated public key**	**Generated private key**	**Key generation duration (N s)**
1	6c560cbf	3c9150ef	10
2	2cbe5280	dd3c45dc	2
3	4c9c05f	779326cf	1
4	fd4b6187	35ab4883	1
5	8eac7f93	cacfd04f	1
6	a9af3aed	15f1d153	1
7	b482a6cf	a24b0245	1
8	c1087c3f	d4f183c5	2
9	87b8b57e	f7d095eb	2
10	f778b303	923179f8	1
11	b300abde	db92348f	1
12	9441c96b	a184e745	1
13	68c48d2b	e28239ad	1
14	c76804a5	3cc0946d	1
15	198c92bf	4df6e4ff	1
16	9b020fc7	abda87ff	1
17	6618af1e	c1af82a7	1
18	baa0576d	69c1ff22	1
19	6d8c4ab1	54267087	2
20	8ea4dd8f	c9137c12	2

**Figure 2:**
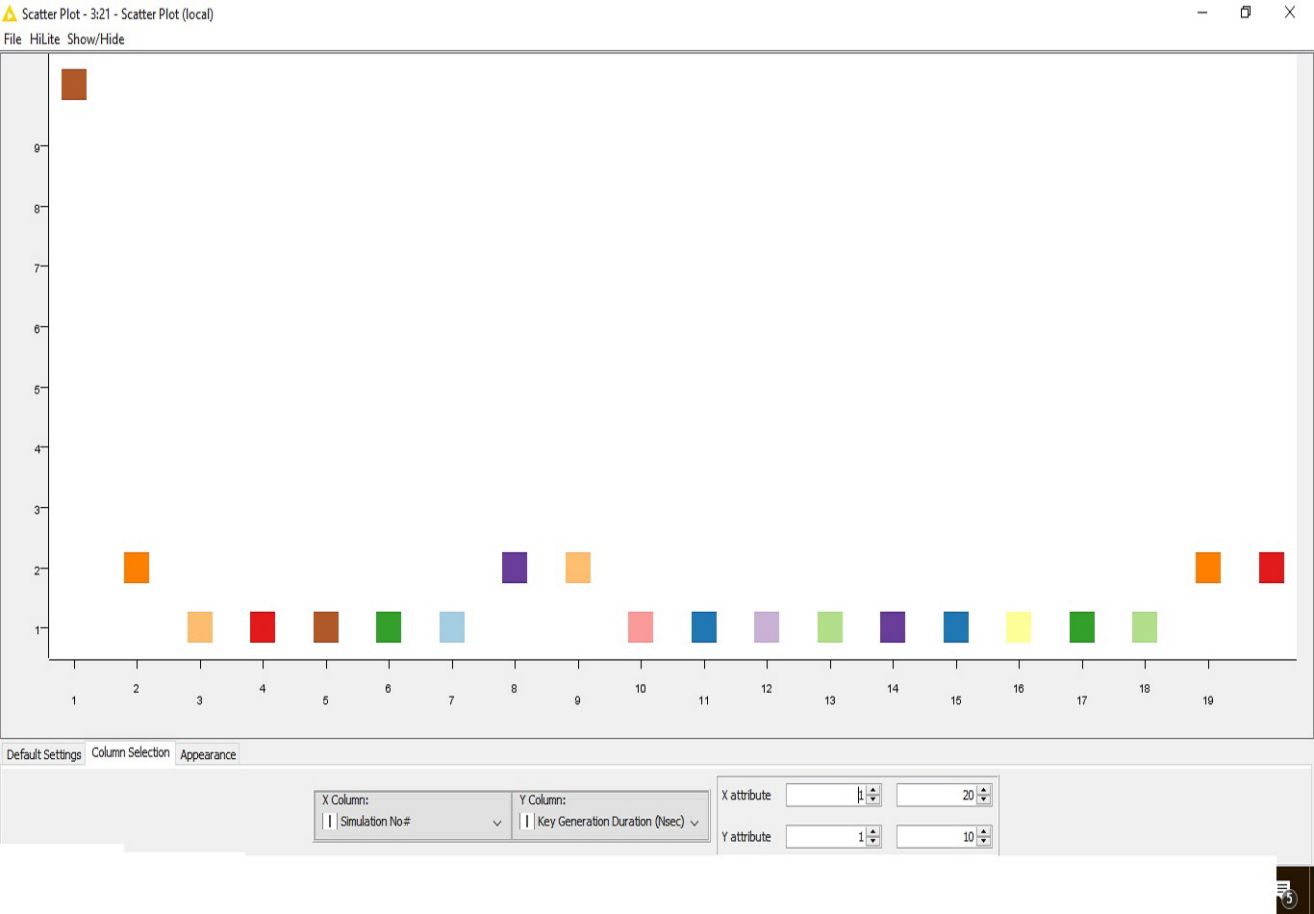
Key generation.

The outcome is also visualized graphically here (Fig. 2).

The time complexity for the key generation is progressively reducing due to the chaos-based implementation of the algorithm.

### Trend analysis for size based time complexity

Thirdly, the time complexity trend is analyzed on different file sizes (Table 3).

**Table 3:** Encryption and decryption time complexity trend analysis.

**File size (KB)**	**Encryption time (N-s)**	**Decryption time (N-s)**
40	40	40
80	80	80
120	120	120
160	160	110
200	170	202
240	240	340
280	280	310
320	320	320
360	360	360
400	400	400
440	440	440
480	480	470
520	520	491
560	533	560
600	600	600
640	640	640
680	680	655
720	720	670
760	760	760

**Figure 3:**
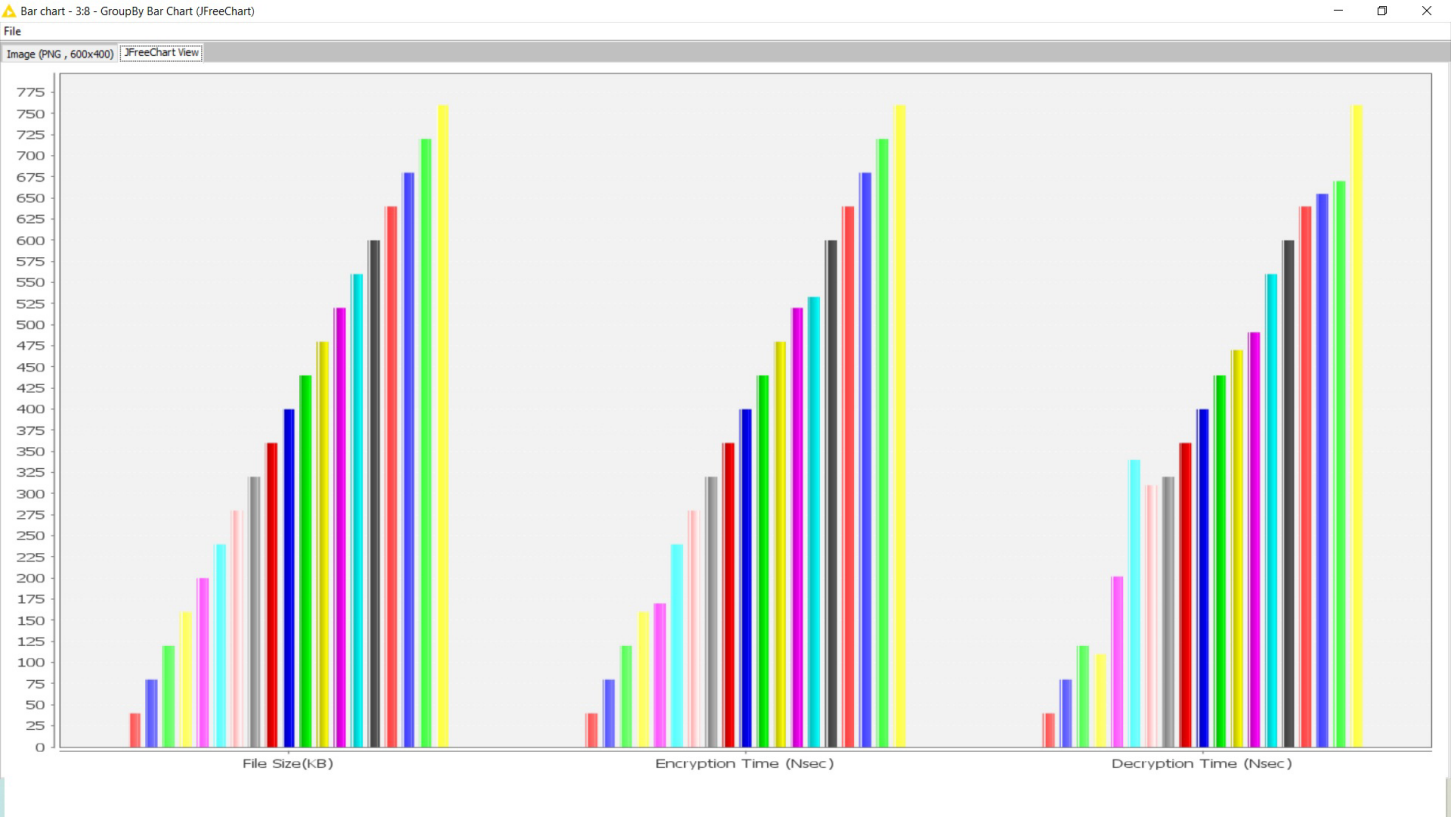
Encryption and decryption time complexity trend analysis.

The outcome is also visualized graphically here (Fig. 3).

The time complexity is a fairly linear trend for any growing file size during encryption and decryption. Hence, a clear conclusion can be generated that the key generation process does not impact the security protocols. Neither relies on the size of the content to be encrypted. It provides a significant observation to be regarded as independent of the security protocols from the contents.

### Cloud deployment results

Fourth, as these proposed algorithms are primarily designed for the cloud-based data security aspects, after the deployment of these algorithms, the performance has been compared (Table 4). During the simulation, two sets of analyses are carried out, the initial one without the security measures for data transmission and the second one with the data transmission measures.

**Table 4:** Cloud deployment performance analysis.

**Observation parameters**	**Time without security (s)**	**Time with security (s)**
VM Selection Mean	0.0005600	0.0006251
VM Selection Std. Dev	0.0039700	0.0040484
Host Selection Mean	0.0002400	0.0003014
Host Selection Std. Dev	0.0005000	0.0005907
VM Reallocation Mean	0.0003300	0.0003926
VM Reallocation Std. Dev	0.0005400	0.0005918

**Figure 4:**
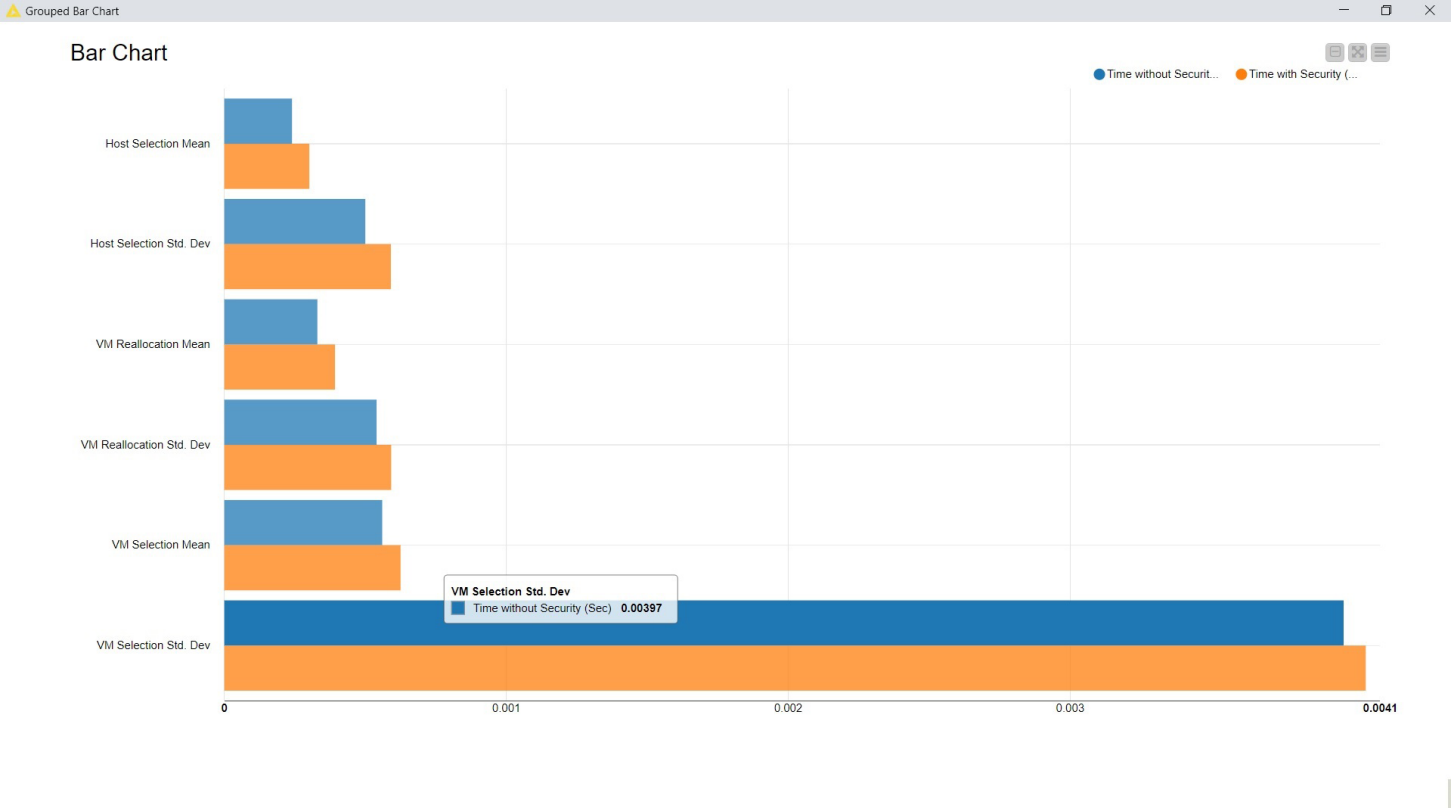
Cloud deployment analysis.

The outcome is also visualized graphically here (Fig. 4).

The fundamental observation from this simulation is that the proposed security measures do not distract from the time complexity beyond 4%. Hence, the computing capabilities utilized for the general data management on the cloud may not be improvised or upgraded. It provides yet another advantage of adopting these proposed security measures.

### Randomness verification results with hypothesis

Fifth, the randomness of the key generation is the most important factor for security key generation. Thus, the true randomness must be tested. In the previous section of this work, the fundamental process for verifying randomness was presented depending on the correlation theory. The proposed verification method is also elaborated. The test results are furnished here (Table 5).

**Table 5:** Randomness verification.

**Hypothesis**	**p-Value**	**Conclusion**
Null Hypothesis: The generated keys do not correlate with each other. Alternative Hypothesis: The generated keys are completely randomly distributed	0.5234	Lower p-Value removes the null hypothesis, and the alternative hypothesis stays.

Thus, the conclusion of the randomness check defines that the generated key values are entirely random.

### Comparative analysis

Sixth, after the detailed analysis of the results in multiple folds, in this sub-section of the work, the proposed algorithm is compared with similar research outcomes (Table 6).

**Table 6:** Comparative analysis.

**Author, Year**	**Primary method**	**Algorithm complexity**	**Applicability to data security**
X. Zhang et al., 2011	Polynomial Based Key Generation	O(n^3^)	Image Data
W. Hong et al., 2012	Side Matching	O(n^2^)	Image Data
V. Manikandan et al., 2018	Machine Learning	O(n^2^)	All Data Formats
L. Xiong et al., 2018	Wavelet Transformation	O(n-log.n^2^)	Image Data
Proposed Method (TPCCSKG & TBSKV)	Chaos Theory	O(2n)	All Data Formats

Henceforth, it is natural to realize that the proposed method has outperformed all the existing data encryption methods and decryptions.

Finally, in the next section of this work, the final research conclusion is presented.

## Conclusion

This work aims to address the decade-long key bottleneck issues for current data encryption methods. Nonetheless, the latest outcomes illustrated the randomness problems of the vital era. This work would henceforth apply chaos-based principles to create spontaneous incidents. Moreover, the encryption keys are produced with randomness using the event sequences. Two-phased key generation is provided to minimize the risk of assaults. A further limiting factor is that core validity was discovered in the parallel study outcomes. The actual key method is dependable when it comes to generating random numbers.

It can be observed that the connection test would lead to reduced accuracy. It is recommended to use a control-based correlation approach to measure the true randomness of the produced keys. Using the chaos principle, the work has a greatly diminished time complexity. If the file size of an encrypted and decrypted portion of the file increases, the time complexity stays constant. Just 2% of this analysis remains to be done in cloud infrastructure settings. In the future, this analysis will represent recent methodological progress in data encryption.

### Future scope of the current work

The following can be listed as future scope of the work:

• The key generation method can be further optimized using the genetic optimization methods.
• The applicability of the key generation method can be tested for adaptation on multi-modal data from various sources.
• The proposed method must be adopted for distributed architecture.

##  Supplemental Information

10.7717/peerj-cs.628/supp-1 Supplemental Information 1 Code

## Additional Information and Declarations

### Data Availability

The following information was supplied regarding data availability: The complete code is available in the Supplementary Files.

## References

[ref-1] Alfalou A, Brosseau C, Abdallah N, Jridi M (2011). Simultaneous fusion compression and encryption of multiple images. Optics Express.

[ref-2] Bender W, Gruhl D, Morimoto N, Lu A (1996). Techniques for data hiding. IBM Systems Journal.

[ref-3] Cox I, Miller M, Bloom J, Fridrich J, Kalker T (2007). Digital watermarking and steganography.

[ref-4] Dworkin MJ (2007). Recommendation for block cipher modes of operation: the CMAC mode for authentication. Special Publication (NIST SP).

[ref-5] Garima B, Abhishek M (2014). Enhanced spread spectrum image watermarking with compression-encryption technique.

[ref-6] Hong W, Chen T-S, Wu H-Y (2012). An improved reversible data hiding in encrypted images using side match. IEEE Signal Processing Letters.

[ref-7] Iwendi CO, Allen AR (2012). Enhanced security technique for wireless sensor network nodes.

[ref-8] Jeeva AL, Palanisamy V, Kanagaram K (2012). Comparative analysis of performance efficiency and security measures of some encryption algorithms. International Journal of Engineering Research and Applications (IJERA).

[ref-9] Johnson NF, Jajodia S (1998). Exploring steganography: seeing the unseen. Computer.

[ref-10] Liu L, Lu Y, Yan X (2019). Polynomial-based extended secret image sharing scheme with reversible and unexpanded covers. Multimedia Tools and Applications.

[ref-11] Manikandan V, Masilamani V (2018). Reversible data hiding scheme during encryption using machine learning. Procedia Computer Science.

[ref-12] Muhammad U, Nordin Z (2016). Chaos-based secure data compression (CSDC).

[ref-13] Ni Z, Shi Y-Q, Ansari N, Su W (2006). Reversible data hiding. IEEE Transactions on Circuits and Systems for Video Technology.

[ref-14] Puech W, Chaumont M, Strauss O (2008). A reversible data hiding method for encrypted images. Electronic Imaging.

[ref-15] Qian Z, Zhang X (2016). Reversible data hiding in encrypted images with distributed source encoding. IEEE Transactions on Circuits and Systems for Video Technology.

[ref-16] Shunmugan S, Arockia Jansi Rani P (2016). Encryption-then-compression techniques: a survey.

[ref-17] Stallings W (2011). Cryptography and network security: principles and practice.

[ref-18] Tang Z-LY, Liu Q, Zhang W, Yu H (2012). A chaos-based joint compression and encryption scheme using mutated adaptive Huffman tree.

[ref-19] Thakur N, Omprakash K (2018). Compression mechanism for multimedia system in consideration of information security.

[ref-20] Weng S, Shi Y, Hong W, Yao Y (2019). Dynamic improved pixel value ordering reversible data hiding. Information Sciences.

[ref-21] Xiong L, Xu Z, Shi Y-Q (2018). An integer wavelet transform based scheme for reversible data hiding in encrypted images. Multidimensional Systems and Signal Processing.

[ref-22] Zhang X (2011). Reversible data hiding in an encrypted image. IEEE Signal Processing Letters.

